# Cost-effectiveness analyses of amivantamab plus lazertinib and lazertinib versus osimertinib in non-small cell lung cancer with EGFR mutations

**DOI:** 10.3389/fphar.2025.1527614

**Published:** 2025-05-02

**Authors:** Hui Zhang, Yueyun Li, Yuhang Liu, Haonan Li, Hong Wang

**Affiliations:** ^1^ School of Medical Business, Guangdong Pharmaceutical University, Guangzhou, Guangdong, China; ^2^ Guangdong Health Economics and Promotion Research Center, Guangdong Pharmaceutical University, Guangzhou, Guangdong, China; ^3^ Guangdong Health Economics and Health Promotion Research Center, Guangdong Pharmaceutical University, Guangzhou, Guangdong, China; ^4^ School of Pharmaceutical Sciences, Peking University, Beijing, China; ^5^ International Research Centre for Medicinal Administration, Peking University, Beijing, China

**Keywords:** amivantamab, lazertinib, osimertinib, cost-effectiveness analysis, non-small cell lung cancer

## Abstract

**Background:**

The combination of amivantamab and lazertinib has demonstrated clinically significant and sustained antitumor effects in both treatment-naïve and osimertinib-pretreated advanced non-small cell lung cancer (NSCLC) patients harboring previously untreated epidermal growth factor receptor (EGFR) mutations.

**Objectives:**

A cost-effectiveness analysis was conducted to compare three therapeutic strategies, namely, amivantamab with lazertinib combination therapy, lazertinib monotherapy, and osimertinib monotherapy, for advanced NSCLC patients with EGFR mutations; the patients included both treatment-naïve individuals and those previously treated with osimertinib.

**Methods:**

Based on a previous multicenter randomized double-blind phase III trial (NCT04487080) for evaluating amivantamab–lazertinib versus osimertinib in EGFR-mutated advanced NSCLC patients (both treatment-naïve and osimertinib-pretreated), we constructed a Markov model for 3-week cycles over a 5-year horizon. The primary outcomes of the model included total costs, quality-adjusted life years (QALYs), and incremental cost-effectiveness ratio (ICER), where all economic parameters were discounted at 3.0% annually. The cost-utility analyses employed China’s *per capita* gross domestic product for 2023 (ranging from $12,295.7 to $36,887.0) as the willingness-to-pay (WTP) threshold supplemented by comprehensive sensitivity and scenario analyses to verify the model robustness.

**Results:**

The economic evaluations demonstrated that compared to osimertinib monotherapy, the amivantamab–lazertinib combination yielded an additional 1.11 QALYs at an incremental cost of $1,342,374, producing an ICER of $1,211,236/QALY that substantially exceeds the $36,887 WTP threshold. Similarly, lazertinib monotherapy showed a QALY gain of 0.71 with $224,248 of additional costs (ICER = $315,640/QALY), also surpassing the lower threshold of $12,296. The sensitivity analysis showed that the predominant model driver was drug acquisition costs.

**Conclusion:**

The economic analyses indicate that neither amivantamab–lazertinib combination therapy nor lazertinib monotherapy represents a cost-effective first-line option for EGFR exon 20 insertion-positive NSCLC compared to osimertinib monotherapy. The substantial drug acquisition costs are the primary contributors to the unfavorable economic profiles of these treatments. Hence, future clinical implementations should carefully weigh the considerable therapeutic benefits against the significant financial burdens to achieve an optimal risk–benefit equilibrium.

## 1 Introduction

In 2022, lung cancers accounted for approximately 2.5 million new diagnoses and 1.8 million deaths globally, representing the most prevalent cause of cancer-related morbidity and mortality. Of these, non-small cell lung cancer (NSCLC) constituted 12.4% of all new cancer cases (1 in 8) and 18.7% of cancer-related deaths (1 in 5), imposing substantial physical, psychological, and socioeconomic burden on the affected individuals as well as their communities and healthcare infrastructures worldwide (Bray et al., 2024a; Xing et al., 2025; Bray et al., 2024b). The emergence of numerous novel medicines has improved the survival outcome of NSCLC. Nevertheless, the prices of these new medicines pose an economic burden for the affected individuals and significantly strains healthcare systems, especially in North America, east Asia, and northern Europe (Bray et al., 2024a).

The epidermal growth factor receptor (EGFR) is the most frequent type of mutation observed in NSCLC, while exon 20 insertions (ex20ins) represent the third most predominant EGFR subtype (Liu et al., 2024; Robichaux et al., 2018a). Although osimertinib (a third-generation EGFR tyrosine kinase inhibitor (TKI)) is used as the primary treatment for EGFR-mutant NSCLC (Johnson et al., 2022), clinical observations reveal two critical limitations: heightened incidence of adverse events (AEs) with third-generation TKIs; diminished efficacy against ex20ins variants owing to conformational alterations of the kinase-active site (Riess et al., 2024; Robichaux et al., 2018b). Consequently, targeted agents designed specifically for EGFR ex20ins (encompassing novel TKIs and bispecific antibodies) have emerged as viable therapeutic alternatives (Shi et al., 2024). Among these novel agents, amivantamab is a dual-targeting antibody that acts on EGFR and mesenchymal–epithelial transition (MET) receptors, demonstrating significant antitumor effects via various pathways through engagement of the FcγRIII receptor that plays a key role. As a fully human bispecific antibody, amivantamab (JNJ-61186372) simultaneously engages EGFR and MET receptors to disrupt oncogenic signaling in NSCLC. Its mechanism includes extracellular domain binding to prevent ligand activation, facilitation of receptor–antibody complex clearance, and induction of immune effector functions like macrophage trogocytosis and natural killer (NK) cell cytotoxicity via Fc-dependent pathways. Fcγ3R is predominantly found on monocytes, macrophages, and NK cells and plays a pivotal role in mediating antibody-dependent cellular cytotoxicity. Amivantamab exerts its therapeutic effects by suppressing EGFR and MET receptor expressions in NSCLC cells, thereby attenuating the downstream signaling pathways (Ye et al., 2021; Wang et al., 2012). The Fcγ3 receptor demonstrates heightened affinity for antibodies containing reduced-core fucose modifications. Structurally, amivantamab incorporates this low-fucose characteristic to facilitate robust binding to Fcγ3R. This interaction not only induces programmed cell death in tumor cells but also potentiates the tumoricidal activities of both macrophages and NK cells (Jarantow et al., 2015; Satoh et al., 2006; Sabari et al., 2021). The cytotoxic activities of immune cells against tumors additionally promote cytokine secretion, resulting in elevated intercellular adhesion molecule-1 (ICAM1) levels. Consequently, EGFR 20 insertion-mutated cancer cells exhibit increased surface CD54 expressions, increasing their vulnerability to cell lysis. As a highly selective third-generation EGFR TKI with central nervous system penetration, lazertinib effectively targets the activation of EGFR mutations and T790M resistance variants; further, its tolerability supports its use in combination therapies (Liu et al., 2024; Lee et al., 2022; Yun et al., 2019; Heppner et al., 2022).

The MARIPOSA intervention was conducted as a global randomized phase 3 trial to compare the efficacy and safety of amivantamab–lazertinib combination therapy against osimertinib monotherapy in previously untreated EGFR-mutated advanced NSCLC patients. The combination therapy group achieved significantly longer median progression-free survival (PFS; 23.7 months) compared to the osimertinib group (16.6 months), with a hazard ratio (HR) of 0.70 (95% confidence interval (CI): 0.58–0.85, *p* < 0.001). This favorable safety profile characterized by mostly mild treatment-related AEs supports the consideration of amivantamab–lazertinib as a first-line therapeutic alternative to osmertinib (Cho et al., 2024). Although the amivantamab–lazertinib combination demonstrates clinical benefits for EGFR-mutated advanced NSCLC, its substantial treatment costs pose significant challenges to patient accessibility and healthcare economic sustainability. This economic burden necessitates a thorough evaluation of the cost-effectiveness of the combination regimen. The present study is a comparative cost-effectiveness analysis between amivantamab–lazertinib combination therapy and osimertinib monotherapy in the MARIPOSA cohort.

## 2 Materials and methods

### 2.1 Target population

The target population and interventions in the present study were identical to those of the MARIPOSA trial. The MARIPOSA trial involved a total of 1,074 patients enrolled from multicenter clinical trial sites across multiple countries and regions, reflecting diverse global healthcare resources. The inclusion criteria for the trial were as follows. First, at least one measurable lesion should meet the RECIST v1.1 criteria (not previously irradiated); if only one measurable lesion was present, a prior diagnostic biopsy was permitted, provided baseline imaging was performed ≥14 d after biopsy. Second, the ECOG performance status should be 0–1. Third, no prior EGFR TKI therapy or systemic treatment should have been provided for stage III/IV disease (adjuvant/neoadjuvant therapy for stage I/II was allowed if completed >12 months before metastasis). Fourth, asymptomatic or treated/stable brain metastases were allowed, and patients with symptomatic brain metastases must have had stable disease for ≥2 weeks before randomization (no steroids or only low-dose steroids ≤10 mg/d prednisone equivalent). The exclusion criteria for the study were as follows: use of investigational drugs within 12 months before randomization; current participation in another clinical trial; active malignancy other than the target disease under study; uncontrolled comorbidities.

### 2.2 Interventions

The MARIPOSA clinical trial (NCT04487080) employed a 2:2:1 randomization scheme for the EGFR-mutant advanced NSCLC patients across three treatment arms. In the combination arm, patients received amivantamab via intravenous infusion (1,050 mg for <80 kg or 1,400 mg for ≥80 kg) during the initial 4-week cycle with split dosing on days 1 and 2, followed by biweekly administration from the second cycle onward, concurrent with daily oral lazertinib of 240 mg. The patients in the control group were administered the standard regimen of osimertinib at 80 mg daily. The monotherapy arm received oral lazertinib at 240 mg once daily. Treatment was maintained until disease progression, unacceptable toxicity occurrence, or patient discontinuation. Subsequent therapies following progression were determined by the investigators in accordance with established clinical guidelines.

The study used standardized baseline parameters, including mean body weight of 70 kg, body surface area of 1.86 m^2^, and creatinine clearance of 70 mL/min (Yue et al., 2024). The safety assessments specifically focused on severe AEs (grade ≥3) that occurred with a frequency of 5% or greater in either the combination therapy (amivantamab–lazertinib) or osimertinib monotherapy group. As all of the study data were exclusively derived from the MARIPOSA clinical trial without additional subject enrollment, separate approval from an independent ethics committee was not required.

### 2.3 Model construction

**FIGURE 1:**
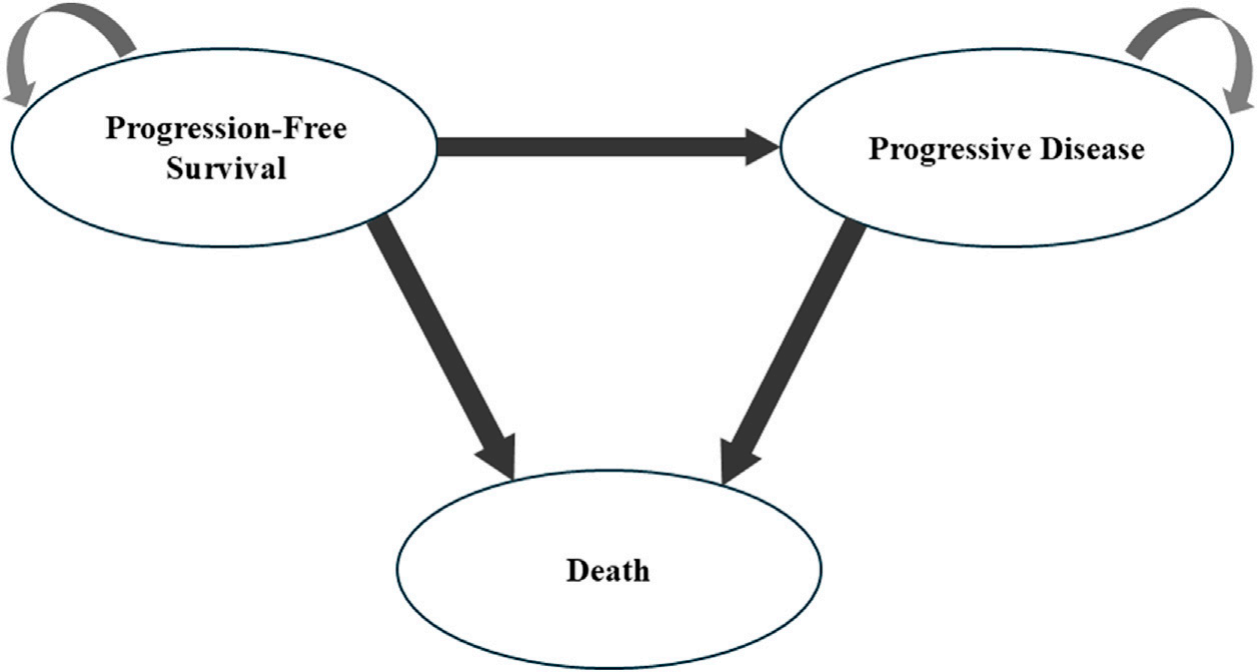
Markov model structure used in the present study.

A Markov model incorporating three distinct health states, namely, PFS, progressive disease (PD), and death, was implemented using Excel 2024 and R software version 4.3.3 to analyze the disease trajectories in advanced cancer cases (Figure 1). For model initialization, all subjects were placed in the PFS state, with unidirectional state transitions evaluated at 21-day intervals. Owing to the absence of overall survival (OS) data for the lazertinib monotherapy, the analysis utilized survival curves identical to those of the osimertinib group as a proxy. Based on follow-up results from the MARIPOSA trial, where the median OS was not achieved in the amivantamab–lazertinib arm, the simulation time horizon was set at 5 years (preliminary analyses confirmed that this duration would capture endpoint events for 99% of the patients). The costs and health outcomes were discounted at an annual rate of 3.0%, with the sensitivity analyses exploring a range of 0%–5%. The key economic endpoints comprised total treatment expenditures, quality-adjusted life years (QALYs), and incremental cost-effectiveness ratios (ICERs). All monetary values were standardized to US dollars as of 2023 (exchange rate: 1 USD = 7.08 CNY) after adjustment via healthcare-specific consumer price indices from 2016 to 2023. The cost-effectiveness benchmarks were defined as 1–3 times the *per capita* gross domestic product (GDP) of China for 2023 ($12,621.19–$37,863.51 USD) by referencing official national statistics. Accordingly, interventions demonstrating ICERs below these thresholds were deemed to be cost-effective. Table 1 shows the complete framework of the cost-effectiveness analysis.

**TABLE 1:** Cost-effectiveness analytical framework.

Category	Intervention 1	Intervention 2
Population	Patients with previously untreated or osimertinib-pretreated EGFR-mutated advanced NSCLC.	
Interventions	Amivantamab–lazertinib	Lazertinib
Comparator	Osimertinib	
Decision-analytical model	Markov model	
Outcomes	Direct medical costs, quality-adjusted life years, and incremental cost-effectiveness ratio	
Disease model	Progression-free state, progressed disease state, and death	
Cycle length	3 weeks	
Time horizon	5 years	
Annual discount rate	3%	
Willingness to pay	1–3 times the forecasted *per capita* GDP of 2023	
Scenario analyses	1: The cost parameter of osimertinib estimated for the health states using the current price is included in the medical insurance in China; 2: The cost parameter of lazertinib was increased by 50% for the health states; 3: The utility values of the base-case analysis were changed.	

### 2.4 Model survival and transition probabilities

From the perspective of China’s healthcare system, this study considered only direct medical costs, including drug costs, follow-up examination costs, end-of-life palliative care costs, and AE management costs (Table 2). The drug costs were based on market prices as of 2023, with amivantamab priced at $17,418.13 per cycle, lazertinib at $13,420.47 per cycle, and osimertinib at $12,623.80 per cycle (Yue et al., 2024). All the other cost parameters, such as follow-up ($542.62) and laboratory tests ($340.20), were derived from published literature (Yue et al., 2024; Ward et al., 2017; Liu et al., 2023; Wu et al., 2019; Liang et al., 2021; Cohen, 2024; Sanders et al., 2016; Hoyle and Henley, 2011) and adjusted to US dollar values as of 2023 using the healthcare consumer price index from 2016 to 2023 (exchange rate: 1 USD = 7.08 CNY).

**TABLE 2:** Model parameters, baseline values, ranges, and distributions for the sensitivity analyses.

Parameter and distribution	Estimated value	DSA		PSA	Source
		range		distribution	
Utility value					
Progression-free survival	0.71	0.57	0.85	Beta	Nafees et al. (2017)
Progressed disease	0.67	0.47	0.71	Beta	Nafees et al. (2017)
Disutility of adverse events					
Paronychia	0.040	0.048	0.032	Beta	Yue et al. (2024)
Rash	0.03	0.02	0.04	Beta	Ward et al. (2017)
Hypoalbuminemia	0.03	0.02	0.04	Beta	Liu et al. (2023)
Increased alanine aminotransferase	0	0	0	Beta	Wu et al. (2019)
Dermatitis acneiform	0.1	0.08	0.12	Beta	Liang et al. (2021)
Pulmonary embolism	0.2	0.16	0.24	Beta	Clinical option
Annual discount rate	3.0%	0	5.0%	Beta	Cohen (2024)
Drug cost per unit ($)					
Amivantamab	17,418.13	13,982.50	20,973.75	Gamma	Yue et al. (2024)
Lazertinib	13,420.47	10,736.38	16,104.56	Gamma	Www.drugs.com
Osimertinib	12,623.80	10,099.04	15,148.56	Gamma	Www.drugs.com
Monitoring cost per unit ($)					
Outpatient	104.41	83.53	125.30	Gamma	Wu et al. (2019)
Best support	3,006.28	2,254.71	3,757.85	Gamma	Wu et al. (2019)
Laboratory test	340.20	272.16	408.24	Gamma	Hoyle and Henley (2011)
Magnetic resonance imaging	95.27	48.9	190.5	Gamma	Sanders et al. (2016)
Follow-up	542.62	406.99	678.31	Gamma	Wu et al. (2019)
End-of-life care	40,708.33	30,531.25	50,885.41	Gamma	Wu et al. (2019)
Adverse event management cost ($)					
Paronychia	9,396.0	7,516.8	11,275.2	Gamma	Yue et al. (2024)
Rash	400.0	320.0	480.0	Gamma	Yue et al. (2024)
Hypoalbuminemia	3,000.0	2400.0	3,600.0	Gamma	Yue et al. (2024)
ALT/AST increase	0	0	0	Gamma	Wu et al. (2019)
Dermatitis acneiform	6.9	5.2	8.7	Gamma	Liang et al. (2021)
Pulmonary embolism	7,055.0	5,644.0	8,466.0	Gamma	Clinical option

The survival analysis data were obtained from the MARIPOSA clinical trial. Then, Kaplan–Meier survival curves were extracted for the amivantamab–lazertinib combination group, lazertinib monotherapy group, and osimertinib group using GetData Graph Digitizer software. The OS and PFS curves of each treatment group were reconstructed using R software (version 4.3.3), and six parametric distributions (log-logistic, log-normal, Weibull, Gompertz, exponential, and gamma) were applied to fit the survival data. The distribution selection prioritized minimal values for both Akaike’s and Bayesian information criteria (AIC and BIC) (Ding et al., 2021). Supplementary Tables S1, S2 present the comprehensive goodness-of-fit metrics, while R-software-derived estimates for the fundamental parameters (shape parameter (λ) and scale parameter (γ)) were obtained through computational analyses. For the health utility values, the utility for PFS was 0.71 and that for PD was 0.67, where both were derived from the Chinese subgroup data of the global NSCLC patient health preference study conducted by Nafees et al. (2017). The utility decrements for the AEs included paronychia (−0.040) and rash (−0.03), and the detailed parameters and distributions are provided in Table 2.

### 2.5 Cost and utility estimates

From the perspective of the healthcare system, our research focused only on the direct medical costs, including the costs of the drugs, AE management, and monitoring. The AEs included paronychia, rash, hypoalbuminemia, ALT/AST increase, dermatitis acneiform, and pulmonary embolism. The treatment cost parameters were primarily extracted from extant publications, and subsequent validation and refinement was conducted through consultations with a panel of 18 board-certified oncologists practicing at tertiary medical centers across three major Chinese regions. The monitoring frequencies followed the guidelines for management of stage III NSCLC according to the American Society of Clinical Oncology (2023) (Daly et al., 2022). The health outcome measurement utilized the QALY parameter, with PFS and PD states assigned utility values of 0.71 and 0.67, respectively, as established in prior research (Nafees et al., 2017). The disutility of AEs was also considered in this model to evaluate the negative effects of the AEs (Yue et al., 2024; Ward et al., 2017; Liu et al., 2023; Wu et al., 2019; Liang et al., 2021; Cohen, 2024). The detailed cost and utility estimates are displayed in Table 2.

### 2.6 Sensitivity analysis

To assess model stability, we conducted both univariate and probabilistic sensitivity analyses. The univariate approach enabled identification of parameters exerting substantial influences on the ICER outcomes and facilitated a thorough evaluation of the parameter-related uncertainties across the entire model; the result interpretation was further enhanced using tornado plot visualizations (Heppner et al., 2022). The probabilistic sensitivity analysis accounted for the parameter uncertainties by applying appropriate probability distributions, namely, the gamma distributions for cost-related variables and beta distributions for health utility measures. Thereafter, extensive Monte Carlo simulations were conducted with 1,000 iterations to assess outcome variability. The results were presented using cost-effectiveness scatter plots and acceptability curves, demonstrating the probabilities of cost-effectiveness for amivantamab–lazertinib combination therapy, lazertinib monotherapy, and crizotinib across varying willingness-to-pay (WTP) thresholds. The ranges and distribution types of the parameters used in the sensitivity analyses are detailed in Table 2.

## 3 Results

### 3.1 Base-case results

Within a 5-year time frame and after applying the discounts, the combination treatment approach with amivantamab and lazertinib demonstrated the greatest average benefit of 2.41 QALYs and incurred the highest average total cost of $1,258,927.53. In comparison, the lazertinib-only strategy was estimated to yield an average of 2.17 QALYs with associated costs of $349,058.51, while the osimertinib strategy resulted in an average of 1.74 QALYs and costs amounting to $1,198,445.02 (Table 3). Our analysis revealed that the amivantamab–lazertinib combination significantly outperformed both lazertinib and osimertinib monotherapies. Specifically, compared to osimertinib, the amivantamab–lazertinib regimen was expected to provide an additional 0.66 QALYs on average, with an incremental cost of $1,258,927.53, leading to an ICER of $90,956.45 per QALY gained.

**TABLE 3:** Cost-effectiveness analysis results for the base case.

Parameters and distributions	Outcomes of the Markov model			Incremental changes (vs. osimertinib)	
	Amivantamab–lazertinib	Lazertinib	Osimertinib	Amivantamab–lazertinib	Lazertinib
Costs	1,977,959.21	859,832.99	635,584.82	1,342,374.38	224,248.17
Drug costs	1,951,220.73	764,298.13	590,890.36	1,360,330.37	173,407.77
Monitoring costs	100,880.51	74,935.33	69,413.91	31,466.60	5,521.42
Adverse event management costs	78,916.68	71,048.27	58,821.25	20,095.43	12,227.02
Overall life years	6.09	5.48	4.34	1.75	1.14
Total QALYs	4.01	3.61	2.90	1.11	0.71
Incremental cost per QALY gained				315,639.51	1,211,235.50

### 3.2 One-way sensitivity analysis

**FIGURE 2:**
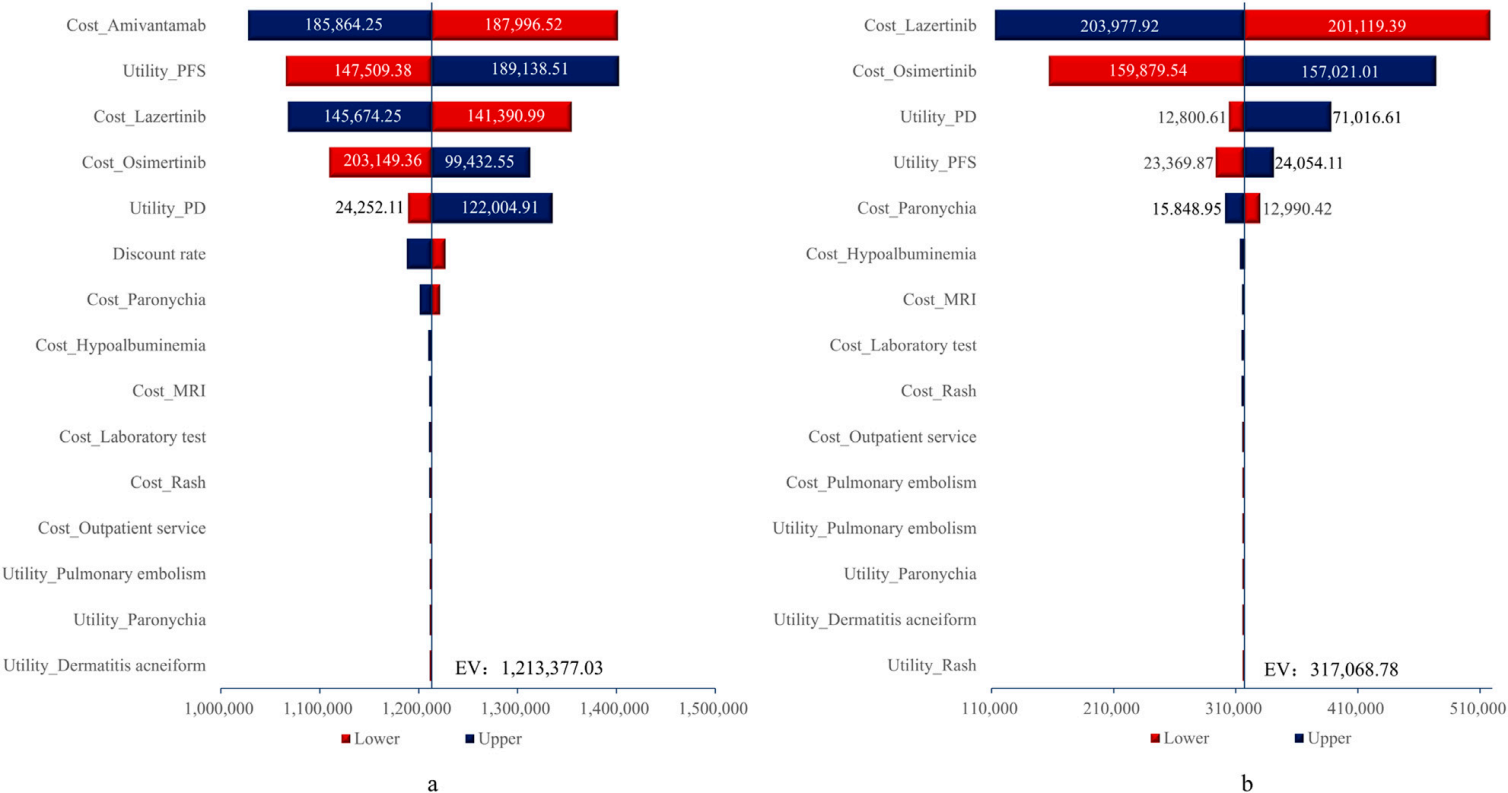
Tornado diagrams for **(A)** amivantamab-lazertinib vs. osimertinib and **(B)** lazertinib vs. osimertinib. EV, expected value.

The tornado diagram for the one-way sensitivity analysis is shown in Figure 2. As illustrated in Figure 2, the parameters with the greatest impacts on the ICERs for the amivantamab–lazertinib combination therapy and lazertinib monotherapy were drug costs (accounting for 98.65% of total costs, with lazertinib comprising 55.82% and osimertinib 42.93%) and utility values for the PFS and PD states. When the costs of amivantamab and lazertinib increased to their upper limits, the ICERs exceeded the WTP threshold based on thrice the value of the *per capita* GDP. For all other parameter variations, the ICER values exceeded the WTP threshold, indicating the robustness of the base-case analysis results.

### 3.3 Probabilistic sensitivity analysis

**FIGURE 3:**
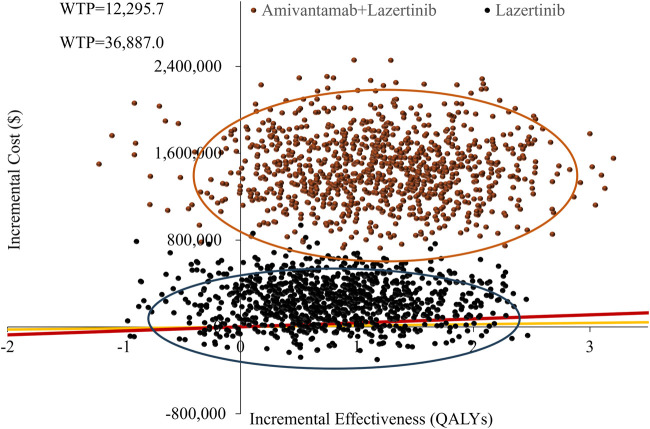
Probabilistic sensitivity analysis results of the different treatments.

**FIGURE 4:**
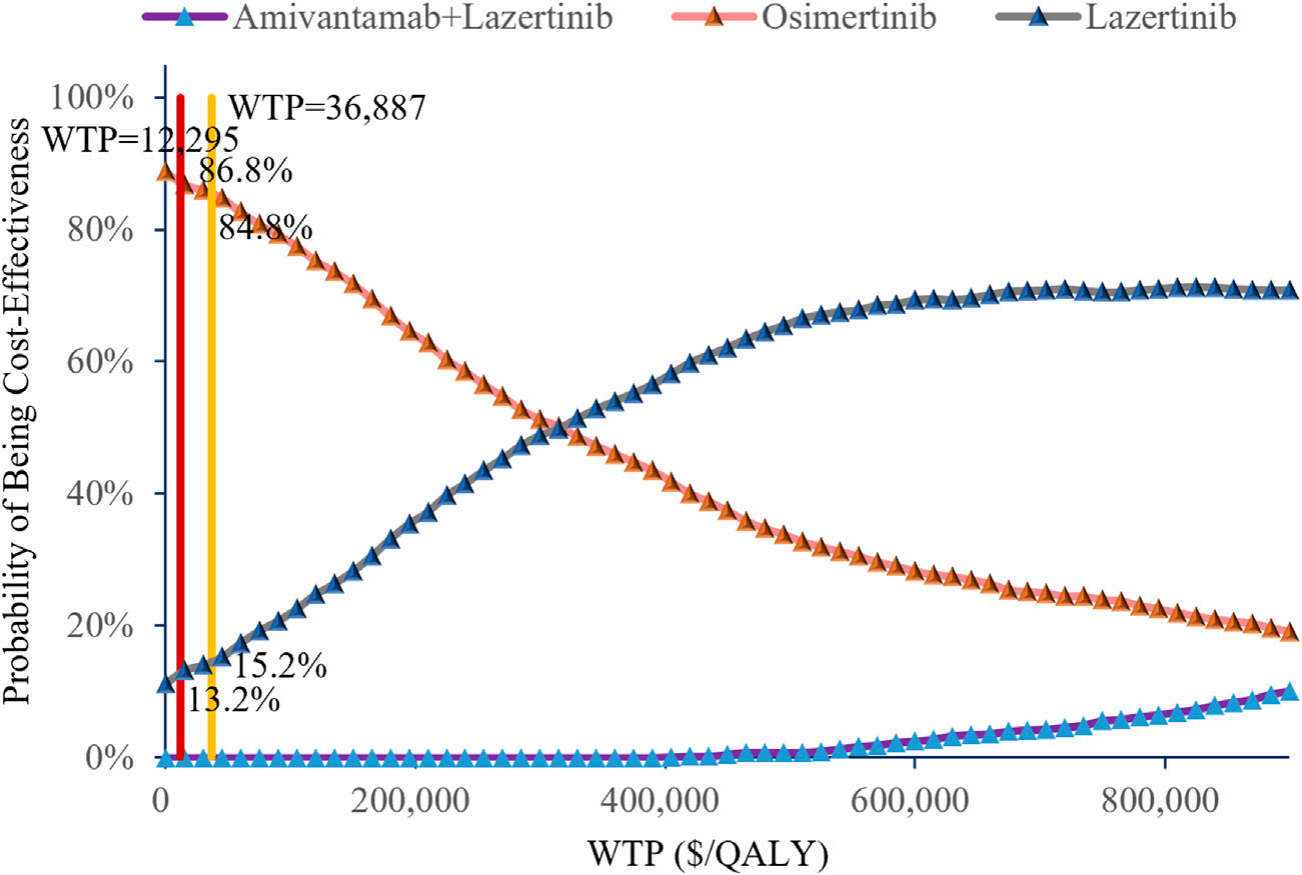
Cost-effectiveness acceptability curves of the different treatments.

The cost-effectiveness scatter plot and acceptability curve from the probabilistic sensitivity analysis are shown in Figures 3, 4, respectively. Figure 3 demonstrates that most of the scatter points are located in the first quadrant, indicating that both amivantamab–lazertinib combination therapy and lazertinib monotherapy have superior clinical efficacies compared to osimertinib monotherapy in treating EGFR-mutated advanced NSCLC. However, from an economic perspective and a comparison of the amivantamab–lazertinib combination therapy with crizotinib regimen, all scatter points are observed to lie above the WTP threshold curve of thrice the *per capita* GDP of China ($36,887/QALY), suggesting that the combination therapy is not cost-effective. For lazertinib monotherapy versus crizotinib, more than half the scatter points are distributed above thrice the GDP threshold, indicating a similar economic inefficiency. The cost-effectiveness acceptability curve in Figure 4 further validates these findings. The results from 1,000 Monte Carlo simulations reveal that at a WTP threshold of $12,295.7/QALY, osimertinib demonstrates 86.8% probability of being cost-effective versus 13.2% for lazertinib. When the WTP threshold is increased to $36,887.0/QALY, osimertinib still maintains an 84.8% cost-effectiveness probability. These consistent results demonstrate that under the current pricing systems, although both amivantamab–lazertinib combination (median PFS of 23.7 months) and lazertinib monotherapy show longer PFS than osimertinib (16.6 months), neither choice meets the cost-effectiveness standards of the Chinese pharmacoeconomic evaluations.

### 3.4 Value of information analysis

**FIGURE 5:**
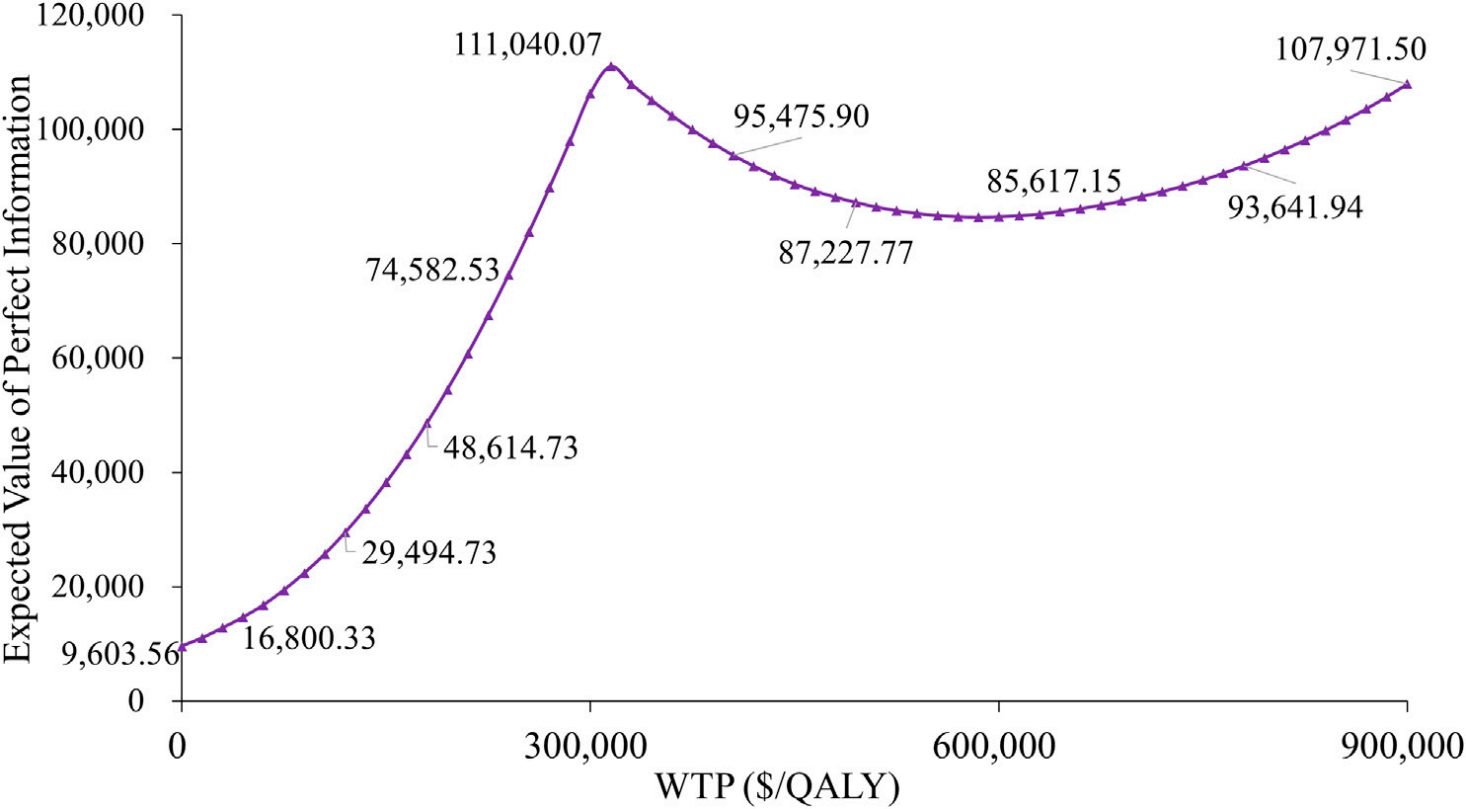
Expected value of perfect information curve.

The expected value of perfect information (EVPI) analysis revealed substantial uncertainty in treatment selection. At a WTP threshold of $300,000 per QALY, the per-patient EVPI was $106,301 (Figure 5), suggesting that additional research could significantly reduce the decision uncertainty. Notably, the EVPI shows threshold dependency; when applying a more conservative WTP threshold of $90,000 per QALY, the per-patient EVPI decreased to $22,380. These results indicate that uncertainty in economic evaluations is particularly sensitive to the cost-effectiveness threshold selected.

### 3.5 Scenario analysis

Scenario analysis is often used to examine the uncertainties of methodologies and models; as such, three scenario analyses were conducted in this study. In scenario analysis 1, the cost parameter of osimertinib estimated for the health states was assumed using the current price included in the medical insurance in China and used as an input parameter to assess the cost-effectiveness of each regimen. In scenario analysis 2, when the cost parameter of lazertinib was increased by 50% for the health states, lazertinib was still found to be the most cost-effective solution. In scenario analysis 3, when the utility values of the base-case analysis changed (Table 3), the amivantamab–lazertinib and lazertinib-only treatments were still dominant compared to osimertinib monotherapy. The changes to the utility values are presented in Table 4, and the corresponding results are presented in Table 5.

**TABLE 4:** Parameters and distributions with changes to the utility values.

Parameter and distributions	Estimated value	DSA		PSA	Source
		Range (±20%)		Distribution	
Scenario analysis 1					
Osimertinib	509.11	407.29	610.93	Gamma	Www.yaozh.com
Scenario analysis 2					
Lazertinib	5840.10	4672.08	7008.12	Gamma	Increased by 50%
Scenario analysis 3					
Progressed disease	0.321	0.257	0.385	Beta	Nafees et al. (2017)
Progression-free survival	0.804	0.643	0.965	Beta	Nafees et al. (2017)

**TABLE 5:** Results of the scenario analyses.

Results of scenario analysis 1			
Group	Estimated cost (osimertinib)	Increase in cost	ICER
Amivantamab–lazertinib (vs. osimertinib)	95,424.43	764,408.56	1,075,939.89
Lazertinib (vs. crizotinib)		1,882,534.78	1,698,626.68

Results of scenario analysis 2			
Group	Estimated cost (lazertinib)	Increase in cost	ICER
Amivantamab–lazertinib (vs. osimertinib)	453,426.87	−182,157.95	−256,395.62
Lazertinib (vs. crizotinib)		893,121.85	805,871.23

Results of scenario analysis 3			
Group	Total QALYs	Increase in QALYs	ICER
Amivantamab–lazertinib (vs. osimertinib)	3.56 (2.53)	1.03	435,917.09
Lazertinib (vs. osimertinib)	3.05 (2.53)	0.51	1,303,323.12

## 4 Discussion

The global cancer mortality data from the World Health Organization (WHO) consistently identifies lung cancer as the predominant contributor to cancer-associated deaths, with the annual mortality figures reaching several million cases worldwide (Barta et al., 2019). Pharmacoeconomic evaluations provide scientific evidence to inform public health policy decisions and healthcare system development by establishing objective and comprehensive evidentiary frameworks. The advent of immunotherapy and targeted therapies has markedly improved survival outcomes for NSCLC patients. Among the various genetic mutations, EGFR mutations are the most prevalent type. TKIs have significantly prolonged the PFS and OS in EGFR-mutant NSCLC patients, with the second- and third-generation TKIs demonstrating enhanced efficacies (Cooper et al., 2022). Approximately 10% of the EGFR mutations involve exon 20 insertions, which typically exhibit poor responses to TKI therapy (Meador et al., 2021). Although current research primarily focuses on TKI-sensitive mutations like exon 19 deletions and L858R point mutations, the novel bispecific antibody amivantamab shows unique therapeutic effects against exon 20 insertion mutations. By simultaneously targeting EGFR and MET receptor pathways in platinum-based chemotherapy-resistant cancer cases, amivantamab demonstrates both direct killing of tumor cells and enhanced cytolytic susceptibility (Billowria et al., 2023).

In this study, we present a cost-effectiveness analysis comparison of amivantamab–lazertinib combination therapy and lazertinib monotherapy against standard osimertinib treatment in advanced NSCLC patients with EGFR exon 20 insertion mutations. The results show that although the amivantamab–lazertinib group achieved 4.01 QALYs, its ICER of $1,211,236/QALY substantially exceeds the WTP threshold of $36,887/QALY. Similarly, lazertinib monotherapy yielded 3.61 QALYs with an ICER of $315,640/QALY, surpassing the lower WTP limit of $12,296/QALY. These findings suggest that neither regimen offers cost-effectiveness advantages over osimertinib.

One-way sensitivity analysis was used to identify amivantamab and lazertinib treatment costs along with the PFS state utility values as the primary determinants of ICER variability. The model robustness was confirmed across plausible parameter ranges. As an innovative EGFR-METR bispecific antibody, the market accessibility and pricing of amivantamab substantially influence its economic profile. Despite demonstrating superior clinical efficacy (median PFS: 23.7 vs. 16.6 months for osimertinib; HR = 0.70, *p* < 0.001) at the current pricing levels, future indication expansions and therapeutic advancements may enable price optimization to improve cost-effectiveness.

We employed a Markov model in this study to assess the cost-effectiveness of first-line amivantamab–lazertinib combination therapy for EGFR-mutated advanced NSCLC. As such, several limitations should be noted: the partitioned survival model utilizes interim OS data from the INSPIRE trial, necessitating parametric extrapolation that may not fully reflect the actual clinical outcomes; real-world payment considerations, such as out-of-pocket expenses by the patients, were not incorporated; potential uncertainties exist regarding both the model structure and parameter sources. These limitations could influence the precision of the economic evaluations for the treatment strategies.

## 5 Conclusion

From the perspective of the healthcare system, both amivantamab–lazertinib combination therapy and lazertinib monotherapy demonstrate inferior cost-effectiveness compared to osimertinib as first-line treatments for advanced NSCLC patients harboring EGFR exon 20 insertion mutations. Although amivantamab exhibits significant efficacy in improving the survival outcomes for NSCLC patients with EGFR-sensitive mutations (e.g., exon 19 deletions or L858R mutations), its substantial treatment costs present a major barrier to broad clinical implementation. Future treatment strategies should therefore emphasize optimizing the cost–benefit ratios of these treatments to ensure sustainable healthcare resource utilization.

## Data Availability

The original contributions presented in the study are included in the article/Supplementary Material, further inquiries can be directed to the corresponding authors.
