# Supplementary material for: Cost-effectiveness analyses of amivantamab plus lazertinib and lazertinib versus osimertinib in non-small cell lung cancer with EGFR mutations
Source: Front Pharmacol. 2025 May 2;16:1527614. doi: 10.3389/fphar.2025.1527614 (PMC12081244; doi:10.3389/fphar.2025.1527614)

# Supplementary Material

## **Table 1 Parametric functions fitting for PFS and OS of amivantamab–lazertinib**

| **Model** | **PFS** | | **OS** | |
| --- | --- | --- | --- | --- |
|  | **AIC** | **BIC** | **AIC** | **BIC** |
| Exponential | 1,411.164 | 1,415.225 | 872.702,4 | 876.763,9 |
| Gamma | 1,404.082 | 1,412.205 | 874.595,5 | 882.718,4 |
| Gen-gamma | 1,406.041 | 1,418.226 | 875.660,8 | 887.845,1 |
| Gompertz | 1,406.979 | 1,415.102 | 873.555,5 | 881.678,4 |
| Weibull | 1,404.076 | 1,412.199 | 874.541,5 | 882.664,4 |
| Log-logistic | 1,404.911 | 1,413.033 | 875.583,9 | 883.706,8 |
| Log-normal | 1,416.373 | 1,424.496 | 879.639,9 | 887.762,8 |

## **Table 2 Parametric functions fitting for PFS and OS of osimertinib**

| **Model** | **PFS** | | **OS** | |
| --- | --- | --- | --- | --- |
|  | **AIC** | **BIC** | **AIC** | **BIC** |
| Exponential | 1,671.494 | 1,675.555 | 979.075 | 983.136,5 |
| Gamma | 1,647.859 | 1,655.982 | 975.331,2 | 983.454,1 |
| Gen-gamma | 1,649.833 | 1,662.016 | 974.240,8 | 986.425,2 |
| Gompertz | 1,657.526 | 1,665.649 | 969.144,2 | 977.267,1 |
| Weibull | 1,648.263 | 1,656.386 | 974.139,1 | 982.262 |
| Log-logistic | 1,648.487 | 1,656.61 | 976.532,1 | 984.655 |
| Log-normal | 1,661.766 | 1,669.889 | 988.991,1 | 977.114 |

## **Table 3 Parametric functions fitting for PFS and OS of lazertinib**

| **Model** | **PFS** | |
| --- | --- | --- |
|  | **AIC** | **BIC** |
| Exponential | 785.411,5 | 788.786,7 |
| Gamma | 773.988,4 | 780.738,9 |
| Gen-gamma | 775.937,9 | 786.063,7 |
| Gompertz | 778.749,6 | 785.500,2 |
| Weibull | 774.401 | 781.151,6 |
| Log-logistic | 774.602,7 | 781.353,3 |
| Log-normal | 776.841,9 | 783.612,4 |

**Table 4 Distribution and parameters of the OS and PFS curves of the three schemes**

| Parameter | Distribution | Value | |
| --- | --- | --- | --- |
| OS (amivantamab–lazertinib) | Expotional | Rate: 0.010,989,9 | |
| PFS (amivantamab–lazertinib) | Weibull | Shape: 1.385,411 | Scale: 0.013,344 |
| PFS (lazertinib) | Gamma | Rate: 0.036,719,1 | |
| OS (osimertinib) | Gompertz | Shape: 0.054,823,95 | Rate: 0.007,0136,2 |
| PFS (osimertinib) | Gamma | Shape: 1.572,129,9 | Rate: 0.072,792,4 |

## **Table 5 Monitoring frequency of iruplinalkib and alectinib**

| Outpatient | Once every 4 weeks |
| --- | --- |
| MRI | Once every 8 weeks |
| Best supportive care | Once every 12 weeks |
| Follow up | Once every 12 weeks |
| End of life | Once |
| Laboratory test | Once every 12 weeks |

## **Figure 1 Comparison of Original PFS Curve and Reconstructed PFS Curve**

## **
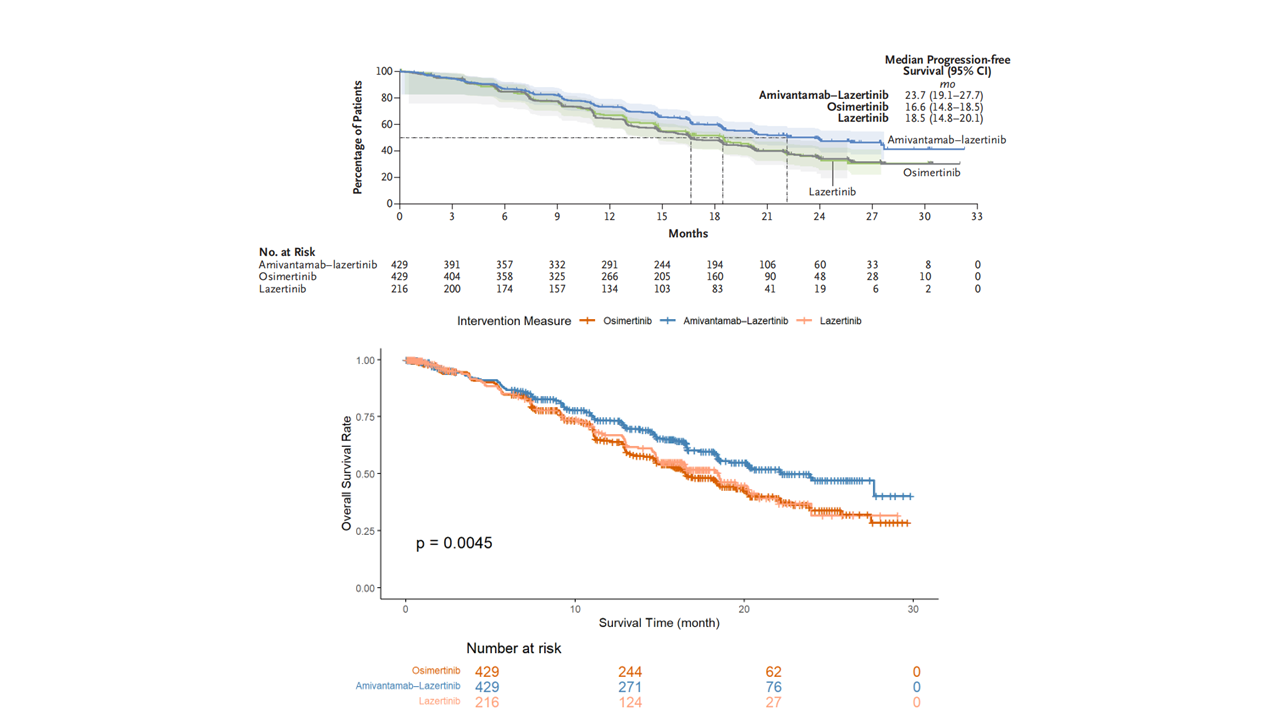
**

## **Figure 2 Comparison of Original OS Curve and Reconstructed OS Curve**


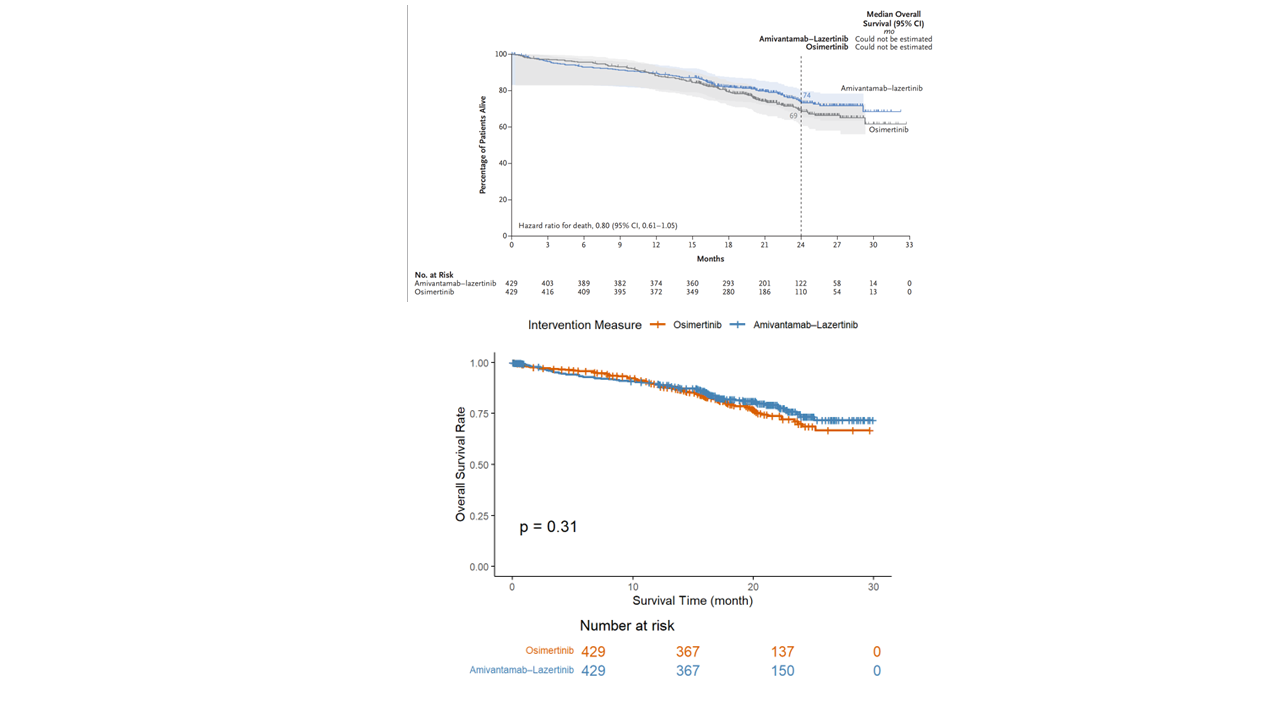


## **Figure 3 KM and parametric survival curve fits for OS of amivantamab–lazertinib**


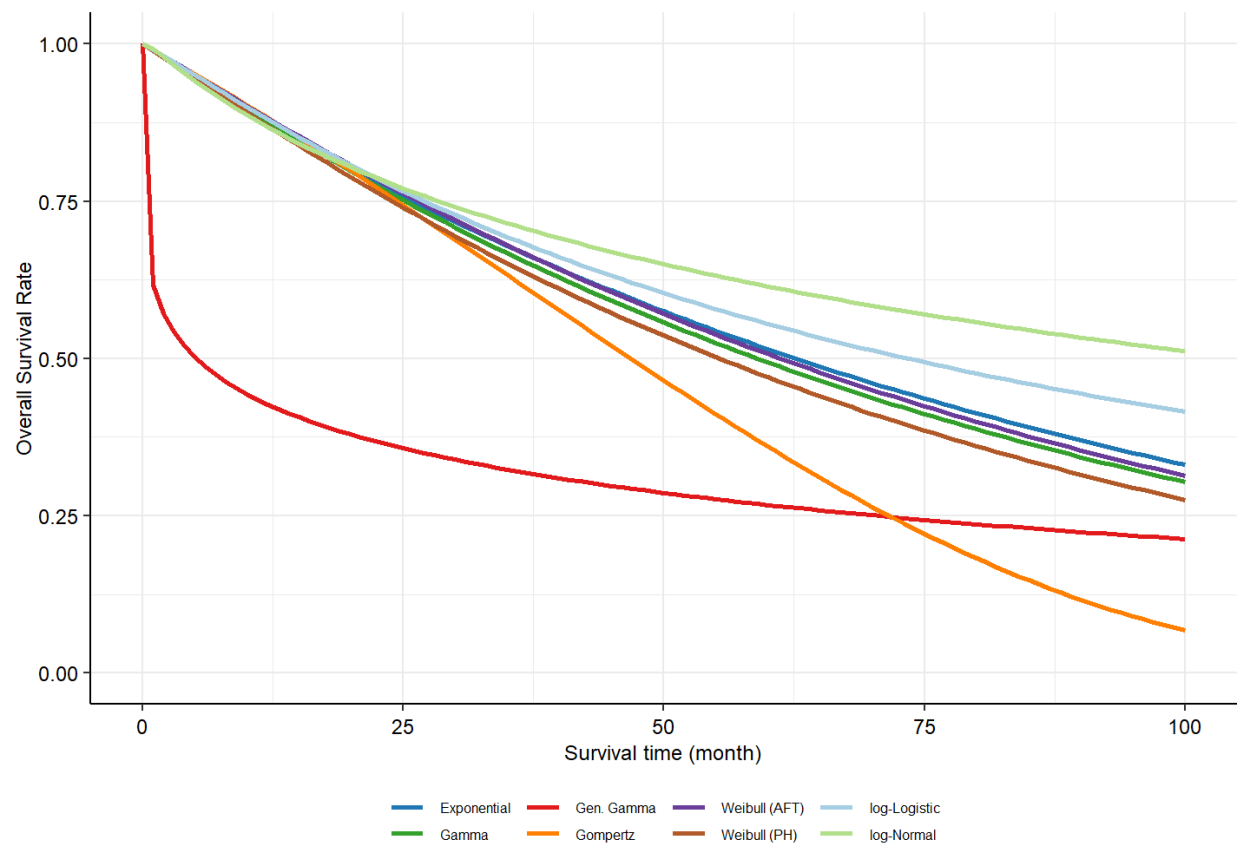


## **Figure 4 KM and parametric survival curve fits for PFS of amivantamab–lazertinib**


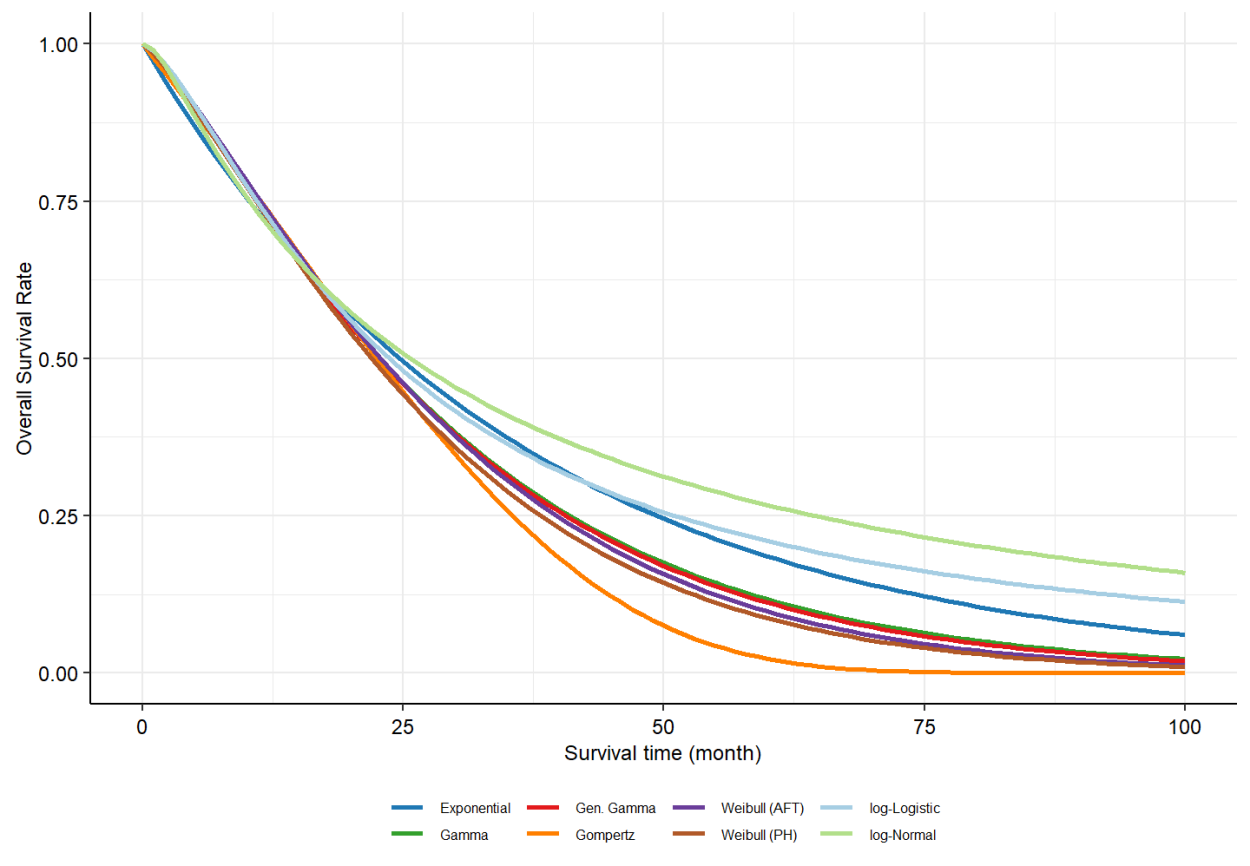


## **Figure 5 KM and parametric survival curve fits for PFS of lazertinib**


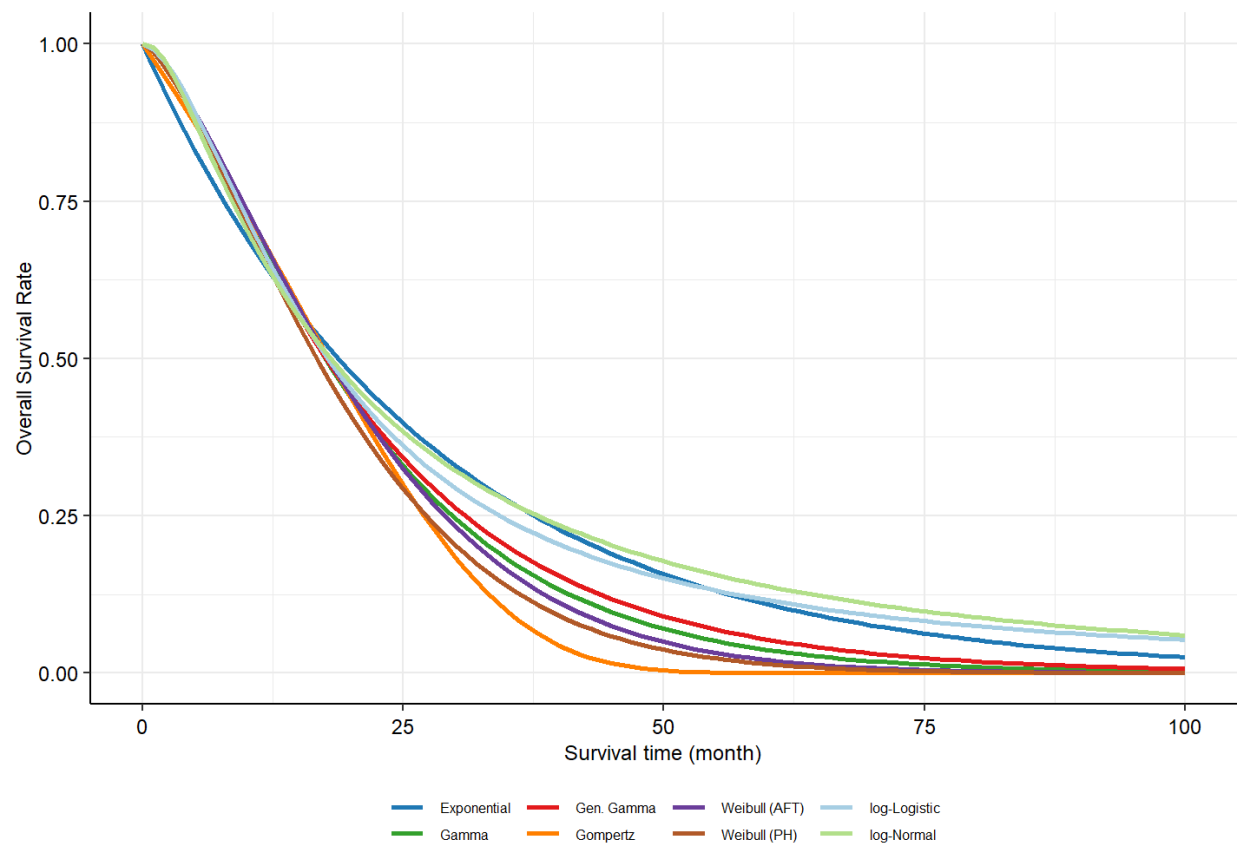


## **Figure 6 KM and parametric survival curve fits for OS of osimertinib**


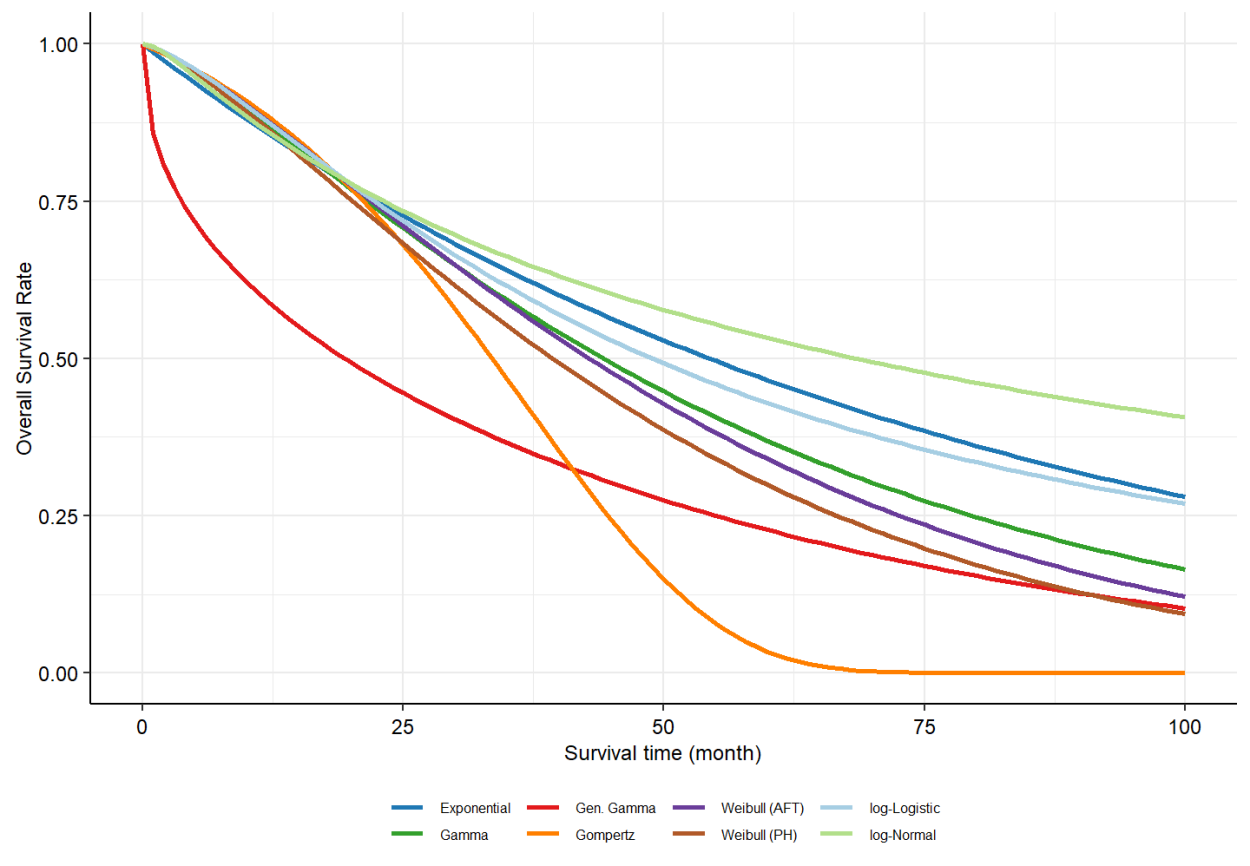


## **Figure 7 KM and parametric survival curve fits for PFS of osimertinib**


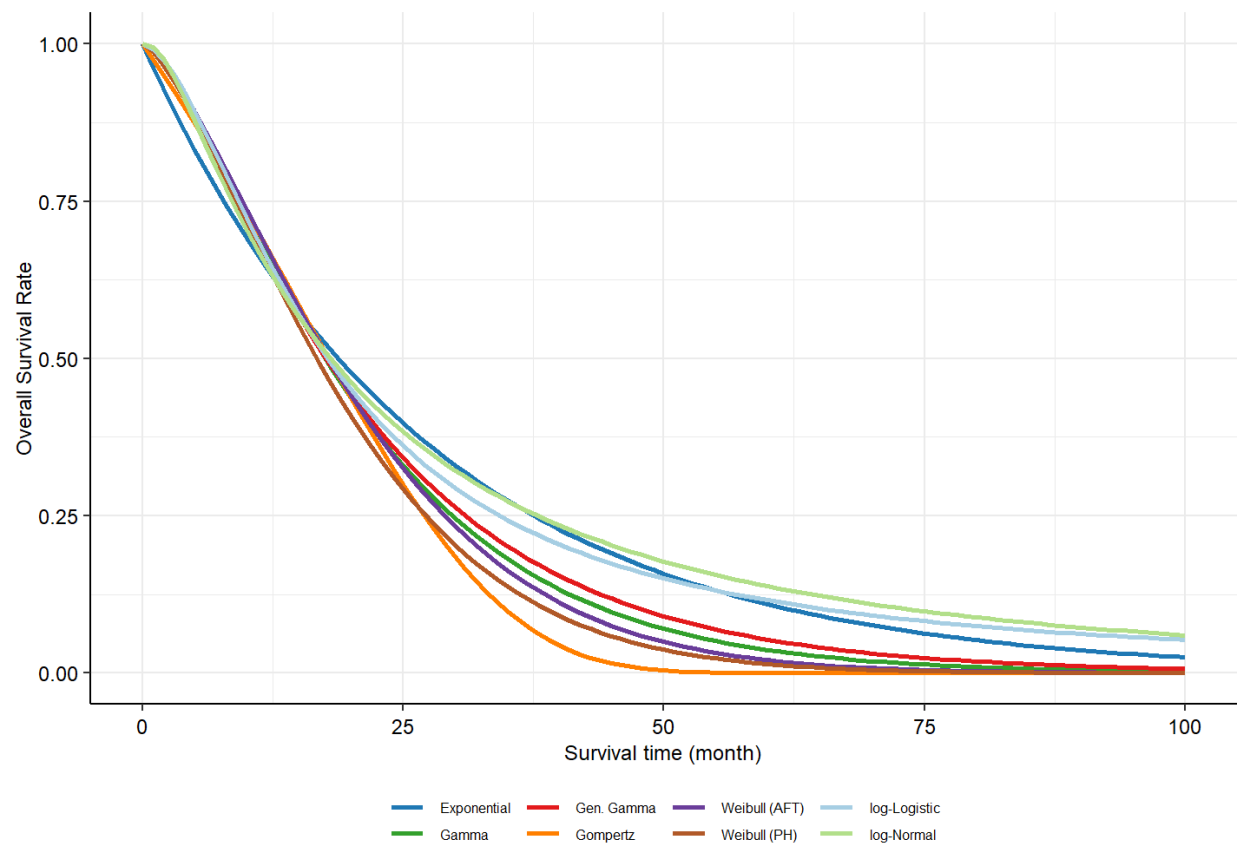

Supplement: Supplementary file 1 [file Supplementaryfile1.docx]
